# Focus on the role of substance P in chronic urticaria

**DOI:** 10.1186/s12948-018-0101-z

**Published:** 2018-11-19

**Authors:** Gino A. Vena, Nicoletta Cassano, E. Di Leo, G. F. Calogiuri, Eustachio Nettis

**Affiliations:** 1 Dermatology and Venereology Private Practice, Bari, Barletta, Italy; 2 Section of Allergy and Clinical Immunology, Unit of Internal Medicine, “F. Miulli” Hospital, Acquaviva delle Fonti, BA Italy; 3 Pneumology Department, Sacro Cuore Hospital, Gallipoli, Lecce Italy; 4 Department of Emergency and Organ Transplantation, School and Chair of Allergology and Clinical Immunology, University of Bari-Aldo Moro, Bari, Italy

**Keywords:** Substance P, Chronic urticaria, Chronic spontaneous urticaria, Neuropeptides, Mast cells, Basophils

## Abstract

**Background:**

Emerging data have strengthened the importance of substance P (SP) as a proinflammatory mediator in human pathology. A role for SP in the pathogenesis of urticaria has long been hypothesized.

**Methods:**

Literature data regarding the possible role of SP in chronic urticaria/chronic spontaneous urticaria (CSU) have been reviewed and summarized in this manuscript. This review is based on pertinent articles that were retrieved by a selective literature search in the PubMed database. Articles in English published up to July 2018 were taken into consideration.

**Results:**

Recent studies in patients with CSU have demonstrated that circulating levels of SP are significantly elevated, in correlation with disease severity, and that SP-positive basophils are upregulated. SP has been shown to trigger degranulation in basophils derived from CSU patients. Moreover, SP can be involved in pseudoallergic reactions and may act as a histamine-releasing factor in a subset of patients with CSU. Current evidence suggests that the biological activity of SP can be exerted not only through the conventional NK-1 receptor but also through the recently identified Mas-related G protein-coupled receptors. MRGPRX2 can cause mast cell activation and has been found to be upregulated in the skin of patients with severe chronic urticaria.

**Conclusions:**

Many findings seem to support the pathogenic involvement of SP in chronic urticaria/CSU. However, further studies are necessary to elucidate the role of SP as a mediator in CSU pathogenesis and a potential new therapeutic target.

## Background

Neuropeptides are short amphipathic peptides that are stored in vesicles and released from peripheral nerve terminals upon calcium influx. Tachykinins are a family of neuropeptides that are involved in a plethora of biological processes and act by binding to the G protein-coupled tachykinin receptors, called neurokinin (NK)-1, NK-2 and NK-3 receptors, with different affinity.

Among members of the tachykinin family, there is substance P (SP), an undecapeptide ubiquitously distributed in the human body. It is secreted from terminals of specific sensory nerves in response to various stimuli, such as leukotrienes, prostaglandins and histamine, and can also be synthesized by lymphocytes, macrophages, eosinophils and dendritic cells [1, 2]. SP selectively binds to NK-1 receptor with higher affinity than to the other two tachykinin receptors.

This neuropeptide modulates many pathophysiological events, such as immunomodulation, antimicrobial host defence, inflammation, pain, itch and cancer [3–6]. It has an important role in the cutaneous neuroimmune network and is recognized as an important pruritogen and a major mediator responsible for neurogenic inflammation in the skin.

## Methods

Literature search was performed in the PubMed database using the terms “urticaria” and “substance P”. Articles in English published up to July 2018 were considered. In order to incorporate relevant data regarding the pathophysiological role of substance P, other pertinent keywords (e.g., “mast cells”, “basophils”) were used in combination with the term “urticaria”. Additional studies were found among the references of the retrieved articles.

### Experimental data on the role of SP in mast cell-mediated cutaneous inflammation

Human skin mast cells are closely associated with sensory nerve endings, which release neuropeptides upon antidromic stimulation by physical or chemical stimuli and stress [7].

These peptides, including SP, are implicated in mast cell activation and degranulation. Many mast cell products, including histamine and tryptase, in turn, may activate sensory nerves, thus supporting the role of a bidirectional crosstalk between mast cells and sensory fibers in mast cell-driven cutaneous inflammation [8].

#### Effects of SP antagonists in cutaneous reactions

In case reports and series, SP antagonists proved to have significant antipruritic effects in drug-induced pruritus, paraneoplastic pruritus, prurigo nodularis, cutaneous T cell lymphoma and brachioradial pruritus [9].

A previous study on the effects of spantide, a competitive inhibitor of SP, suggested the involvement of this neuropeptide in immunological contact urticaria and in the reaction to tuberculin, while no influence was noted on the cutaneous reaction to benzalkonium chloride, UVB irradiation and benzoic acid. In this study, spantide was injected intracutaneously into the volar aspect of the forearm prior to the challenge [10].

The first-generation receptor antagonists were large size peptides and required to be injected intracutaneously. In a more recent study, the effect of aprepitant, a non-peptide SP antagonist, on delayed and immediate cutaneous reactions was evaluated [11]. The results showed that topically applied aprepitant penetrates the skin but does not inhibit patch test and prick test reactions or the associated itch.

#### SP receptors on mast cells

In addition to the high affinity IgE receptor (FcεRI), mast cells express numerous G protein-coupled receptors. The endogenous receptor for SP, NK-1 receptor, is expressed on mast cells and plays a role in IgE and non-IgE-mediated responses in mice. Although NK-1 receptor antagonists are effective regulators of experimental allergic and inflammatory responses in mice, they have not yet proved to have a high therapeutic value as anti-allergic and/or anti-inflammatory agents in humans, suggesting the existence of more relevant alternative signalling pathways [12, 13].

Emerging evidence suggests that SP can mediate some inflammatory events and induce itch through Mas-related G protein-coupled receptors in addition to NK-1 receptor [13].

A new human Mas-related G-protein-coupled receptor expressed on human mast cells (MRGPRX2) was recently found to be activated by basic secretagogues, including SP and drugs associated with pseudo-allergic reactions, although this was shown in the human LAD2 mast cell line [14].

#### Activation of mast cells and basophils

SP causes plasma leakage, vasodilation and increased vascular permeability and evoked immediate dose-dependent wheal, flare and itch responses accompanied by granulocyte infiltration in the skin upon intradermal injection [15–18]. Two pathomechanisms appear to be involved in such reactions: a direct effect on the microcirculation, with induction of vasodilation and plasma extravasation, and also an indirect one through histamine release from skin mast cells [16, 17]. SP is, in fact, a well-known non-immunological stimulus for histamine release.

As recently summarized on the basis of the available evidence [19], SP can induce the degranulation of mast cells in vitro and can increase the responsiveness of mast cells to triggering signals [20–22].

Instead, the results concerning SP-mediated cytokine production by human mast cells appear to be contradictory. Reasons responsible for such conflicting findings may be related to the different experimental conditions and mast cell subtypes examined in the various studies [23].

More specifically, Gibbs et al. [24] found that human skin mast cells were capable of rapidly secreting pro-inflammatory cytokines like tumor necrosis factor (TNF)-alpha and interleukin (IL)-8 following IgE-dependent activation and stimulation by SP, as well as stem cell factor and compound 48/80. In contrast to other types of human mast cells, however, human skin mast cells were incapable of secreting IL-4, IL-5 or IL-13 under such experimental circumstances.

Using in situ hybridization and reverse transcriptase polymerase chain reaction, Okayama et al. [25] showed that mRNA for IL-4, IL-5 and TNF-alpha was induced by immunological activation (e.g., cross-linkage of FcεRI receptors) on human skin mast cells, but only TNF-alpha mRNA was selectively induced by SP.

On the other hand, Guhl et al [23]. reported that the expression of cytokine mRNA in human skin mast cells was not altered by SP. In fact, unlike stimulation via FcεRI receptors, stimulation by SP did not influence the production of TNF-alpha or IL-8 in purified human skin mast cells. In this setting, human skin mast cells appeared to be responsive to SP in a selective manner by eliciting degranulation and subsequent histamine release without the induction of cytokines. Concentration of IL-6 was instead reduced in the supernatant upon SP triggering while being unaltered at the mRNA level, suggesting a post-transcriptional suppressive mechanism.

The involvement of SP in basophil degranulation and accumulation has also been highlighted [26]. SP proved to be a potent chemoattractant for human basophils in vitro acting via NK-1 receptors in basophils and through the engagement of phosphodiesterases and phosphoinositide-3 kinases in the downstream signalling pathway [27].

### Studies specifically addressing chronic urticaria

Chronic urticaria is defined by the presence of repeated episodes of wheals, angioedema or both for more than 6 weeks [28]. Chronic spontaneous urticaria (CSU) is characterized by the spontaneous occurrence of lesions, without any identifiable cause in the majority of cases.

Autoimmune/autoreactive mechanisms have been established to induce CSU in up to nearly 50% of patients because of the presence of histamine-releasing antibodies against FcεRI and less commonly IgE. The triggers for mast cell activation in the remaining cases are unknown, although it has been hypothesized that they might include IgE antibodies against autoallergens, activated complement, and neuropeptides such as SP [29].

Mast cell lines lacking FcεRI have been shown to be activated by sera from CSU patients, suggesting the presence of serum factors responsible for mast cell degranulation through FcεRI-independent mechanisms, at least in a subgroup of patients with CSU [30].

#### Circulating levels of SP

Tedeschi et al. [31] evaluated the serum levels of SP by means of an enzyme immunoassay in 117 patients with CSU, 40 atopic subjects (20 with allergic rhinitis and 20 with allergic asthma) and 24 normal subjects. In vivo and in vitro assessment for histamine-releasing factors, by means of autologous serum skin test (ASST) and basophil histamine release (BHR) assay, respectively, was performed in all CSU patients. Mean serum SP concentration was not significantly different between CSU patients (221.94 ± 17.28 pg/ml) and normal subjects (290.29 ± 41.36 pg/ml). Moreover, no difference in serum SP levels was revealed between patients who were positive and those who were negative on ASST or on BHR assay. Mean serum SP level was significantly higher in the whole group of atopic subjects and in the subgroup of patients with allergic rhinitis than in CSU patients.

In the authors’ opinion, the absence of increased serum SP levels in the majority of CSU patients appeared to deny an essential role of SP as a histamine-releasing factor in CSU in general, without however completely excluding a possible involvement. For instance, the absence of raised serum level might also be due to the release of low levels only at cutaneous level, to the rapid inactivation or alternatively to the prompt binding to mast cell receptors. Nevertheless, in the study by Tedeschi et al. [31], very high levels of serum SP (from 910 to 1100 pg/ml) were observed in 3 of 75 patients positive on ASST and negative on BHR assay, suggesting that SP may be actively implicated in the pathogenesis of CSU in occasional cases.

Evaluation of SP serum concentration in CSU patients was the purpose of another study carried out by Metz et al. [19], who collected samples from 30 healthy subjects, 118 CSU patients and 20 patients with cold urticaria. A significant increase in SP levels was found in patients with CSU (491 ± 24 pg/ml) compared to healthy controls (105 ± 28 pg/ml). Patients with cold urticaria showed also increased levels (280.3 ± 24 pg/ml), but significantly lower than in CSU patients. The discrepancy between these results and those previously reported by Tedeschi et al. [31] might be explained by differences in the blood processing and storing modalities that could have influenced degradation of SP, and possibly also by differences in other methodological factors or in the criteria used for selection of CSU patients, including disease severity [19, 26].

Moreover, Metz et al. [19] were able to demonstrate the direct correlation between SP concentration and disease activity defined by the urticaria activity score (UAS7). Very high levels of SP (> 800 pg/ml) were absent in healthy subjects and in patients with mild CSU and were found in every fourth patient with severe CSU. SP levels did not show significant differences depending on the presence/absence of specific variables, i.e., angioedema, stress as triggering/exacerbating factor, association with anxiety and depression disorders, underlying etiology of CSU [19].

A more recent study confirmed the increase of circulating SP in patients with CSU [26]. In particular, plasma SP level in 15 CSU patients was approximately 3.5-fold higher than the level in plasma of 15 control subjects. The same study disclosed that the percentage of basophils expressing SP and NK-1 receptor were markedly elevated in blood from CSU patients in comparison with control subjects. A positive correlation between the absolute number of basophils and the level of SP (P < 0.001) was observed in peripheral blood of CSU patients.

Another study evaluated the effects of H1-antihistamines on serum levels of selected neuropeptides, including SP, in chronic urticaria [32]. In total, 22 patients were treated with cetirizine 10 mg/day and 16 patients with loratadine 10 mg/day for 7 days. No significant difference was detected between pre- and post-treatment levels of SP, whereas a significant effect on serum levels was observed for other neuropeptides in terms of either a decrease (stem cell factor, neuropeptide Y, vasoactive intestinal peptide, nerve growth factor) or an increase (calcitonin gene-related peptide) after antihistamine therapy.

#### Cutaneous response to SP and role in histamine release

Pseudoallergic reactions against natural food components have been implicated in the elicitation and maintenance of chronic urticaria in some patients. A study enrolled 33 patients with chronic urticaria and pseudoallergic reactions to food, and skin biopsy specimens from such patients were investigated for in vitro mast cell histamine release. Significantly elevated levels of total histamine and spontaneous histamine release were found in patients versus control subjects. In vitro histamine release was not caused by tomato extract itself but was enhanced by the combination of tomato distillates with SP and complement 5a (C5a) but not by anti-IgE, underlining the involvement of IgE-independent mechanisms in pseudo-allergic reactions [33].

Patients with chronic urticaria were found to exhibit enhanced reactions to intradermally injected neuropeptides, such as SP and vasoactive intestinal peptide, as compared to non atopic control subjects. SP-induced wheal and flare reactions were demonstrated to be suppressed by a potent H1 blockade [34].

The cutaneous response to SP was evaluated in 9 patients with CSU and 9 patients with delayed pressure urticaria compared to 9 healthy adults [35]. In response to intradermally injected SP, CSU patients exhibited significantly enhanced and longer lasting wheal reactions, as well as significantly larger and longer lasting flare response as compared to controls. In patients with delayed pressure urticaria, SP-induced wheal and flare responses were intermediate in magnitude compared to the other two patient groups. Responses were examined after a single dose of 20 mg cetirizine, 4 mg dimethindene or placebo, according to a double-blind, placebo-controlled, crossover, randomized assessment. In healthy subjects, SP-induced flares were significantly suppressed only by cetirizine, while both cetirizine and dimethindene affected SP-induced wheal and flare responses in the two patient groups.

SP might act as a possible histamine-releasing factor in a subset of patients with CSU.

Given that SP activates human mast cells via MRGPRX2, Fujisawa et al. [36] studied MRGPRX2 expression in the skin of patients with severe chronic urticaria and found it upregulated as compared to the healthy subjects. The skin-derived cultured mast cells, unlike lung-derived cultured mast cells, expressed MRGPRX2 on the cell surface. Histamine release from the skin-derived cultured mast cells triggered by SP was found to be independent of the traditional SP receptor NK-1 receptor. Instead, SP-induced degranulation and prostaglandin D2 generation in skin-derived cultured mast cells were significantly reduced in MRGPRX2-silenced mast cells. In human skin mast cells, MRGPRX2 was shown to be the responsible receptor for histamine release induced not only by SP, but also by eosinophil peroxidase and major basic protein.

The relationship between basophils and SP in CSU patients was recently explored, with particular emphasis on the ability of SP to cause basophil degranulation [26]. Once added, SP induced up to 41.2% net histamine release from basophils derived from CSU patients, which was comparable with that provoked by anti-IgE, calcium ionophore and *N*-formyl-methionyl-leucyl-phenylalanine. SP-induced histamine release in this setting appeared to be mediated by NK-1 receptor and to occur through a non-cytotoxic mechanism. SP, anti-IgE, calcium ionophore, and *N*-formyl-methionyl-leucyl-phenylalanine also caused histamine release from basophils of healthy control subjects, though to much less extent.

## Conclusions

Many findings seem to support the pathogenic involvement of SP in urticaria, based on the ability of SP to elicit itch and wheal-and-flare response in the skin, to induce mast cell and basophil degranulation and to act as a mast cell sensitizer, enhancing the responsiveness of mast cells to activating triggers [21, 22, 29, 33]. Moreover, recent studies in CSU patients compared to controls have shown increased circulating levels of SP [19, 26], in apparent correlation with disease severity [19], as well as elevated percentages of circulating SP-positive basophils [26]. The upregulation of MRGPRX2 expression in the skin of patients with severe chronic urticaria has also been disclosed [36].

SP may have an important role in pseudoallergic reactions and has been hypothesized to act as a possible histamine-releasing factor in a subset of patients with CSU, especially in those with evidence of histamine-releasing factors different from functional autoantibodies.

The identification of MRGPRX2 on mast cells and the involvement of such receptors in SP-induced activation of human skin mast cells opens new opportunities for understanding pathomechanims of CSU and mast cell-mediated skin disorders and for exploring new therapeutic interventions.

Further studies are needed to clarify the role of SP as a mediator in CSU pathogenesis and a potential new therapeutic target.

## Abbreviations

NK: neurokinin
SP: substance P
FcεRI: high affinity IgE receptor
MRGPRX2: Mas-related G-protein-coupled receptor expressed on human mast cells
TNF: tumor necrosis factor
IL: interleukin
C5a: complement 5a
CSU: chronic spontaneous urticaria
ASST: autologous serum skin test
BHR: basophil histamine release
UAS7: urticaria activity score
